# Pain Neuroscience Education Versus Biomedical Pain Education with Exercise in Primary Dysmenorrhea: A Randomized Controlled Trial

**DOI:** 10.3390/healthcare13161954

**Published:** 2025-08-09

**Authors:** Büşra Nur Erol, Ceren Gürşen, Sezcan Mümüşoğlu, Serap Özgül

**Affiliations:** 1 PhD Program of Physical Therapy and Rehabilitation, Graduate School of Health Sciences, Hacettepe University, Ankara 06100, Türkiye; 2 Department of Therapy and Rehabilitation, Vocational School of Haymana, Ankara University, Ankara 06860, Türkiye; 3 Faculty of Physical Therapy and Rehabilitation, Hacettepe University, Ankara 06100, Türkiye; ceren.gursen@hacettepe.edu.tr (C.G.); serap.kaya@hacettepe.edu.tr (S.Ö.); 4 Department of Obstetrics and Gynecology, Faculty of Medicine, Hacettepe University, Ankara 06230, Türkiye; sezcanmumusoglu@gmail.com

**Keywords:** patient education, menstrual pain, diaphragmatic breathing, stretching exercise, relaxation exercise

## Abstract

**Background/Objectives:** Although pain education is a very important component of chronic pain management, its effects on primary dysmenorrhea (PD) have not been investigated. The aim of this study was to compare the effects of pain neuroscience education (PNE) or biomedical pain education (BPE) combined with exercise training (ET) in PD. **Methods:** Individuals with PD were randomly assigned to PNE or BPE combined with ET (stretching and relaxation exercises) for two menstrual cycles. The PNE focused on pain neurobiology, central sensitization, and brain pain-modulation mechanisms, while the BPE included anatomical and biomechanical explanations of pain. Assessments were conducted at the start of the study, after the intervention period, and at the one-month follow-up after the intervention. The primary outcome measure was menstrual pain (mean and maximum pain) intensity, while the secondary outcome measures included menstrual stress, central sensitization symptoms, and pain catastrophizing. **Results:** All the individuals assigned to the PNE+ET (*n* = 19) and BPE+ET (*n* = 19; control) groups were included in the analysis. PNE or BPE with ET led to improvements in all outcome measures (*p* < 0.05). Furthermore, PNE was found to reduce menstrual pain, central sensitization symptoms, and pain catastrophizing more after the intervention and at follow-up compared to BPE (*p* < 0.05, Cohen’s d = 0.683–1.174). However, menstrual stress decreased at similar levels in both groups (*p* > 0.05). **Conclusions:** The combination of PNE or BPE with ET was demonstrated to be an effective approach for the management of menstrual pain and stress in PD. Furthermore, PNE appears to be more efficacious in addressing menstrual pain, symptoms of central sensitization, and pain cognition compared with BPE. Further studies could investigate the combination of PNE with different education parameters and physiotherapy methods to manage PD.

## 1. Introduction

Primary dysmenorrhea (PD) is characterized by the presence of spasmodic and painful cramps in the lower abdomen that occur just before and/or during menstruation, in the absence of any macroscopic pelvic pathology. In individuals with PD, pain is frequently accompanied by a range of physical and emotional symptoms [1,2].

Although the prevalence of PD varies depending on the region and population, it can reach up to 97.4% and negatively affect various aspects of quality of life [3,4]. Furthermore, dysmenorrhea can potentially lead to the development of chronic pelvic pain over time [5,6]. Therefore, effective pain management strategies for PD can not only provide short-term pain relief, but also prevent the development of central sensitization and chronic pain in the long term [7].

Pharmacological and non-pharmacological approaches are used in the initial steps of PD management. The long-term use of pharmacologic agents is limited by the risk of adverse effects [1,8]. Exercise training (ET) constitutes a primary non-pharmacological approach in PD [9]. Diaphragmatic breathing exercises have been shown to modulate pain by enhancing vagal activity within the autonomic nervous system [10,11] while stretching exercises have been demonstrated to activate endogenous pain modulation and increase local pelvic blood flow through biomechanical and viscoelastic responses in muscle–tendon and neural units [12,13].

Patient and/or pain education is a fundamental element in disease and/or pain management [14]. Biomedical pain education (BPE) is a conventional pain education model that employs a biomedical perspective and aims to provide information regarding normal and damaged tissues, mechanical deviation from normal/expected movement patterns, and tissue degeneration [15,16]. In the traditional biomedical model, pain is considered a direct consequence of tissue damage or structural pathology, and interventions generally focus on eliminating the physical cause or healing the damaged tissue. In this model, the causes of pain are directly attributed to somatic causes, while psychological or social factors are generally ignored [15,16,17]. However, given the biopsychosocial nature of pain, this approach may be inadequate, especially in cases where the structural basis is unclear, such as chronic pain. Indeed, a large proportion of patients with chronic pain experience severe and persistent pain that cannot be explained by objective tissue damage [17,18]. Therefore, instead of a biomedical model that focuses solely on biological factors, more comprehensive approaches that address pain as a multidimensional issue are needed [17].

Pain signals originate in the periphery and are transmitted to the brain via the spinal cord and brainstem; they are processed not only at the spinal cord level but also in higher centers [17,18,19]. Brain regions such as the anterior insula and periaqueductal gray matter (PAG) play a key role in the perception and modulation of pain. The anterior insula integrates the emotional and attentional dimensions of pain, while the PAG mediates brain-derived descending inhibitory pathways to suppress pain [19]. These processes demonstrate that pain is a complex experience shaped by cognitive and emotional factors, as well as physical ones [17,20]. The biopsychosocial model recognizes that pain is not merely a biological process and is influenced by various factors, including the person’s psychological state, behavioral patterns, and social environment [17]. Pain neuroscience education (PNE) is a modern pain management approach that was developed based on this model; it focuses on pain management beyond pain reduction, addressing lifestyle factors and aiming for biopsychosocial change. During the process of change, pain and pain mechanisms are explained, negative thoughts and feelings about pain (e.g., pain catastrophizing) are managed [21,22], and a more active and healthy lifestyle is encouraged [23]. PNE is an intervention method aimed at understanding the biological and neurophysiological bases of pain [17]. PNE teaches individuals that pain is not solely caused by tissue damage; it is shaped by the brain and nervous system’s response to the perception of a threat [18,22]. Because fear-avoidance behaviors can increase the intensity and duration of pain, PNE aims to break this cycle. In this respect, PNE is not only a cognitive intervention but also a holistic educational approach that contributes to the reorganization of biological processes [17]. Inflammatory mediators and neuroinflammatory processes are known to play an important role in pain generation in PD. Increased levels of prostaglandins (PGF2α and PGE2) in the endometrium trigger myometrial contractions and vasoconstriction, exacerbating ischemic pain. Prostaglandins also act as neuromodulators that facilitate pain transmission at nerve endings [24]. Furthermore, levels of proinflammatory cytokines such as TNFα, IL-6, and IL-1 have been shown to be higher in women with PD, and it has been suggested that these cytokines potentiate the pain mechanism by increasing prostaglandin synthesis [24,25]. These molecules are thought to activate a signaling network that increases pain intensity at both the peripheral and central levels [24]. Therefore, biopsychosocial-based approaches such as PNE may have the potential to modulate not only perceptual, but also nervous system responses associated with these biochemical mechanisms. Early pain management with this perspective has been reported to reduce the risk of transition to chronic nociplastic pain [26]. Moreover, it has been documented that PNE, when integrated with exercise or other therapies, provides greater benefits for chronic pain compared to PNE alone [27,28,29].

There have been a number of studies comparing the efficacy of PNE and BPE in the management of pain. The findings of these studies indicated that PNE may be more effective than BPE in reducing pain intensity, symptoms of central sensitization, and pain catastrophizing [16,21,30,31,32,33,34]. However, other studies have reported that PNE and BPE produce similar outcomes [15,21,30,32,33,34,35,36,37]. To the best of our knowledge, there is no research addressing the effects of BPE or PNE in PD. Therefore, the aim of this study was to compare the effects of PNE and BPE, given together with ET, in individuals with PD. Our study hypothesis was that PNE would reduce pain intensity, menstrual distress, central sensitization symptoms, and pain catastrophizing more than BPE in individuals with PD.

## 2. Materials and Methods

### 2.1. Trial Design

The study was designed as a parallel-group randomized controlled trial. The study protocol was approved by the local clinical research ethics committee (decision number: 2022/15-02) and is registered in ClinicalTrials.gov (NCT06040866). The study results are reported in accordance with the Consolidated Standards of Reporting Trials (CONSORT) guidelines [38].

### 2.2. Participants

The study population comprised nulligravid volunteers aged 18 years and older who had been diagnosed with PD by a gynecologist based on the Primary Dysmenorrhea Consensus Guideline [1]. All individuals referred to the physiotherapy and rehabilitation department were informed about the study’s protocol, which is in accordance with the Declaration of Helsinki. Signed informed consent was obtained from all participants prior to their involvement in the study. The inclusion criteria for both groups were as follows: the absence of any known diseases, a regular menstrual cycle (28 ± 7 days), and a menstrual pain intensity ≥ 4 [39], according to a visual analog scale (VAS), in the previous six months and during the most recent menstruation. Patients were excluded from the study if they had any of the following characteristics: a history of pelvic or abdominal surgery; were taking antidepressants, anxiolytics, or oral contraceptive agents; utilized alternative treatments in the previous year; or were using intrauterine contraceptive devices.

### 2.3. Randomization

The randomization process (for lists and group assignments) was conducted by a researcher (S.Ö.) who was not involved in the evaluation and intervention processes. Furthermore, the randomization lists were accessible only to this researcher. Individuals who met the inclusion criteria and gave informed consent were randomly assigned to one of two study groups using an online tool (https://www.randomizer.org/; block size of six, allocation ratio of 1:1). Therefore, in this 2-group study with equal allocation and a block size of 6, 3 individuals in each block were assigned to PNE+ET and 3 individuals to BPE+ET (control group), and the order of these 6 assignments was random. Lists with 4 blocks in two separate groups with a fixed block size of six (6 × 4 = 24 individual assignment lists in each group) were automatically created.

### 2.4. Interventions

Assessments were conducted on the day following the end of the participants’ first menstruation cycle after the start of the study. The PNE+ET group received structured and standardized neuroscience-based pain education sessions and an exercise program. The BPE+ET group (control group) received structured and standardized biomedical-based pain education and the same exercise program as the other group. The interventions began on the first working day after the assessments. The interventions were administered during the non-menstrual period (with the exclusion of the initial 3 days, during which bleeding or discomfort was particularly pronounced) for a total of 2 menstrual cycles, concluding with the onset of the third menstruation. The interventions were executed by a physiotherapist (B.N.E.) who possesses 9 years of experience in this domain, while the outcome measurements were completed by the participants under the supervision of an independent clinician. The researcher who administered the intervention (B.N.E) was not present at the assessments. The clinician was unaware of which intervention was being administered to the individual, and her supervision only consisted of explaining how to complete the scales and checking that the scales were completed.

#### 2.4.1. Stretching and Relaxation Exercises

The participants were given a program that included stretching and diaphragmatic breathing exercises accompanied by soothing instrumental music under the supervision of a physiotherapist in the clinic once a week. Additionally, they were asked to perform the same program at home twice a week. Individual compliance with the exercise sessions was meticulously documented by the physiotherapist, and the compliance rate (number of sessions performed/number of recommended sessions) was calculated.

**Figure 1:**
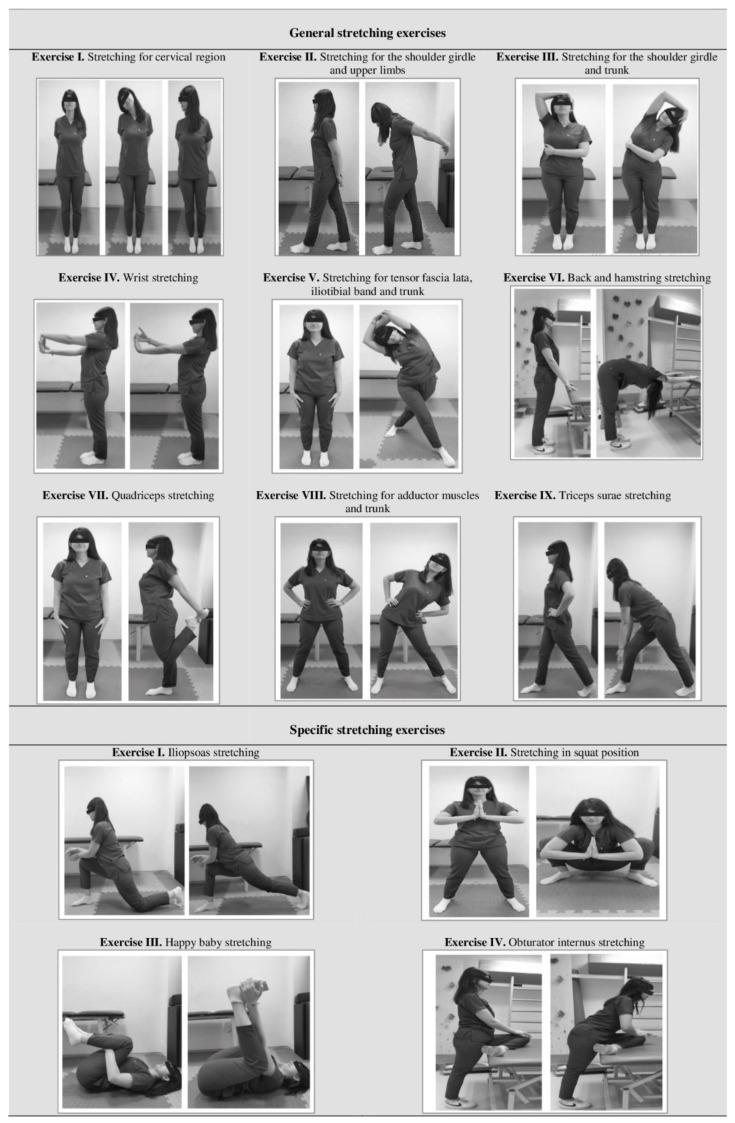
General and specific stretching exercises.

A stretching exercise program was developed based on both the literature and clinical knowledge [40,41,42,43]. The program consisted of a total of 13 active stretching exercises, encompassing both general exercises (Figure 1, exercises I–IX) and pelvis-specific exercises (Figure 1, exercises I–IV). Each stretching exercise session had a duration of approximately 20 min. During each exercise, the participants were instructed to move slowly until they first perceived a stretching sensation [44], pause for 10 s at this point, and then gradually return to the initial position. Each exercise was performed with three repetitions. A brochure containing visual representations and explanatory text was provided to all participants.

As a relaxation exercise, diaphragmatic breathing was performed for a period of 10 min following a series of stretching exercises. During the relaxation phase, the participants were positioned in a semi-recumbent position with knees bent and pillows under the arms and knees in a quiet, warm, temperature-controlled, and dimly lit room. They were instructed to inhale deeply through the nose for approximately four seconds, inflate their abdomen, and then exhale slowly and deeply for about eight seconds through the mouth as if blowing out a candle. With each inhalation and exhalation, they were instructed to further release regional body muscles (head, neck, and shoulders, arms, hands, upper trunk, waist, abdomen and pelvis, thighs, and legs and feet) and focus on the moment [11].

#### 2.4.2. Pain Education

In addition to stretching and relaxation exercises, the participants attended a total of two individual, face-to-face, interactive sessions of either PNE or BPE during the first two weeks of the intervention period. Each session, held once per week, lasted 60 min (45 min of presentation and 15 min of discussion). While the BPE presentation incorporated visual aids, the PNE presentation utilized a combination of narratives, examples, and metaphors. Informational brochures [2,16,17,45,46] compatible with the content of the training sessions were distributed to the participants. The training sessions were repeated with a reminder session at the end of the second menstrual cycle. In addition, both groups were informed about the importance of sleep hygiene, physical activity and exercise, and breathing and relaxation techniques [30]. To ensure standardization of the content and presentations in the pain education sessions, structured presentations and scripts based on the literature were used, and training sessions were conducted according to a predetermined protocol [2,16,17,45,46]. The researcher conducting the training (B.N.E.) was trained by a researcher experienced in PNE and BPE prior to the study.

- Biomedical Pain Education

In the BPE, the participants were informed about pain and PD with a biomedical focus; explanations regarding the mechanisms of pain were not provided. The topics covered included the reproductive system, the menstrual cycle (ovarian and endometrial cycle), the definition of acute/chronic pain and PD, the symptoms and effects of PD, biomedical treatments for PD (medications, TENS, thermal agents, massage, acupuncture, and taping) and their side effects, and lifestyle recommendations (recommendations regarding stress, smoking, genital hygiene, rest, sleep, nutrition, and physical activity) [2,16,20].

- Pain Neuroscience Education

There were no anatomical or biomedical explanations in the PNE. The subjects were taught the physiological, biological, and psychological processes in the experience of pain with a biopsychosocial focus. The topics included pain as a valid and genuine sensory and emotional experience, the brain’s interpretation of pain, peripheral and central sensitization, descending modulation, and the role of immune and endocrine activation [17,45,46]. The program emphasized that pain is independent of tissue damage and that there are six fundamental concepts that inform our understanding of pain: (1). it is the brain’s decision to produce pain; (2). pain is not related to the severity of tissue damage; (3). pain is influenced by factors such as thoughts, activity, sleep hygiene, and stress; (4). the purpose of acute pain is to alert the person to danger or injury; (5). chronic pain is caused by an overstimulated nervous system and no longer has a warning function; and (6). numerous methods (e.g., physical activity, exercise, relaxation, stress management, and good sleep hygiene) can reduce pain and positively affect the quality of life by reducing the heightened sensitivity of the nervous system [45].

### 2.5. Outcomes

#### 2.5.1. Descriptive Outcomes

A comprehensive set of demographic (age, education level, marital status, and employment status), physical (body weight and height), lifestyle (smoking, alcohol consumption, and regular exercise habits), and menstrual (age at menarche, menstrual cycle characteristics, menstrual length, menstrual pain severity and duration, and use of analgesics or anti-inflammatory drugs for menstrual pain) characteristics were documented for the study participants. The criterion for regular exercise habits was determined as exercising for at least 150 min at a moderate intensity or at least 75 min at a vigorous intensity per week [47].

#### 2.5.2. Outcome Measures

Outcome measurements were performed at three time points: after the first menstruation, after performing the intervention for two menstrual cycles (after 3rd menstruation), and 1 month after the end of the interventions (after 4th menstruation).

- Primary outcome measure

*Menstrual pain intensity*

The intensity of menstrual pain experienced by each participant was evaluated using a horizontal Visual Analog Scale (VAS) ranging from 0 to 10 (0: no pain; 10: worst pain imaginable) [48]. The participants were asked to indicate the highest menstrual pain intensity they had experienced during their last menstrual cycle (one day before menstruation and on the first, second, and third days of menstruation) on the VAS. The mean of the scores from each participant for these four time points was calculated to determine the “mean pain intensity” value. The highest of these values was documented as the “maximum pain intensity” value. The VAS has been demonstrated to be a reliable and valid method for measuring experimental and clinical pain and to be sensitive to minor changes in pain intensity [49].

- Secondary outcome measures

*Menstrual stress level*

The severity of menstrual symptoms was evaluated using the Menstrual Distress Questionnaire (MDQ) (r = 0.71–0.97) [50,51]. The questionnaire included 47 symptoms, with the total score ranging from 0 to 188. A high score is indicative of an increase in the perceived level of menstrual stress [51].

*Central sensitization symptoms*

The symptoms of somatic and emotional related to central sensitization was assessed using the Central Sensitization Inventory (CSI) (Cronbach’s α = 0.92; ICC = 0.93; r = 0.34–0.73) [36,52]. The total score ranges from 0 to 100, with higher scores denoting an increase in symptom intensity [36].

*Pain catastrophizing level*

The levels of menstrual pain catastrophizing experienced by the participants were assessed using the Pain Catastrophizing Scale (PCS) (Cronbach’s α = 0.90; r = 0.75–0.90) [53,54]. The total score ranges from 0 to 52, with a higher PCS score indicative of an increased tendency towards catastrophizing [53].

### 2.6. Sample Size

The sample size calculation was performed using G*Power version 3.1 based on the results of studies that used PNE for rotator cuff repair or exercise training in PD that demonstrated clinically significant changes in pain intensity based on a VAS [34,55,56]. In the one-way hypothesis design, it was determined that a total of 32 participants, with 16 participants in each group, should be included in the study to reach 80% power, with a calculated effect size difference (Cohen’s d) of 0.9 and α of 0.05 for type 1 errors. Assuming a 20% attrition rate during the study, a total of 38 participants were included in the study.

### 2.7. Statistical Methods

The data were analyzed with SPSS (Statistical Package for the Social Sciences, version 23, IBM Corporation) software. Descriptive statistics are presented as the mean ± standard deviation or as a percentage. The conformity of the numerical variables to a normal distribution was examined using analytical and visual methods. The independent groups *t*-test or Mann–Whitney U test was used for comparisons between independent groups. The within-group time-dependent variation in the numerical data was analyzed using the Friedman test, and the source of the periodic differences was analyzed using the post hoc Conover test. The relationships between the categorical variables were evaluated using the Fisher–Freeman–Halton exact test. The statistical significance level was set at *p* ≤ 0.05. The effect sizes were calculated using Cohen’s d, with 0.2 indicating a small effect size, 0.5 indicating a medium effect size, and ≥0.8 indicating a large effect size [57].

## 3. Results

**Figure 2:**
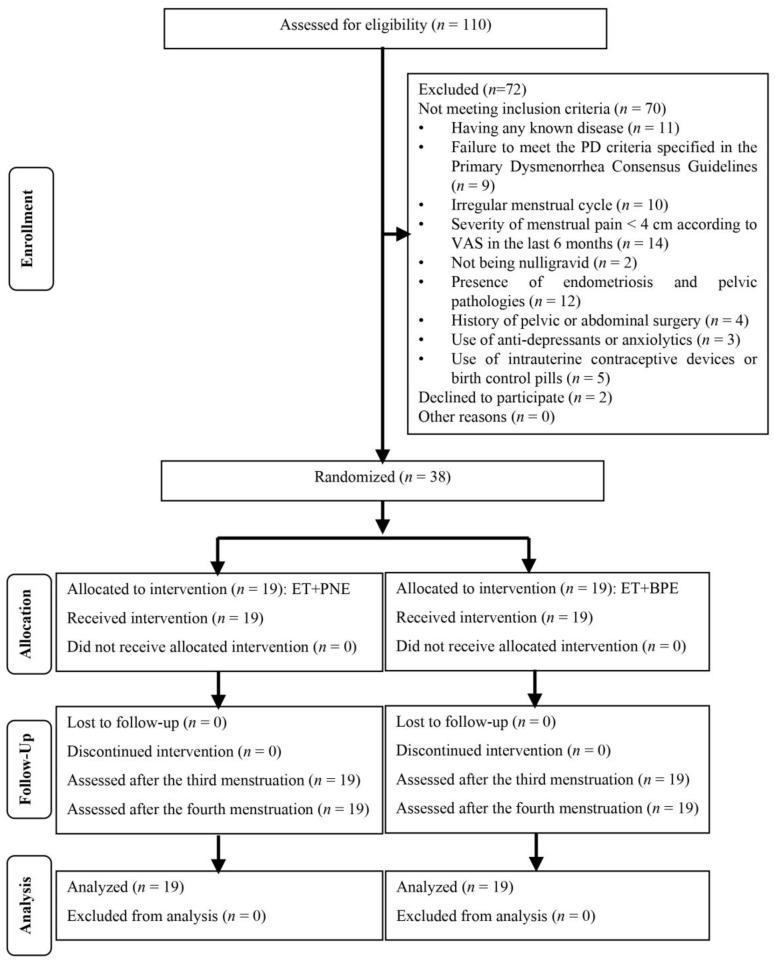
Flowchart of the study. ET: exercise training; PNE: pain neuroscience education; BPE: biomedical pain education.

From October 2023 to November 2024, 110 women were screened for their suitability for participation in the study. Following this screening, 70 individuals were found to be ineligible, and 2 individuals declined to participate. Consequently, 38 volunteers completed the study, with 19 allocated to the PNE+ET group and 19 allocated to the BPE+ET group. No participant withdrew from the study during the intervention period (Figure 2).

The study groups were found to be similar regarding their descriptive characteristics and baseline values for the primary and secondary outcome measures (*p* > 0.05) (Table 1 and Table 2).

All the participants demonstrated full participation in the exercise and pain education sessions at the clinic. The level of compliance with clinical and home exercise sessions was 97.70 ± 4.27% in the PNE+ET group and 97.04 ± 5.65% in the BPE+ET group (*p* > 0.05) (Table 3). During the study period and at the one-month follow-up, no exercise-related adverse effects were reported by the participants. At the one-month follow-up evaluations, all the participants from both groups reported that they had not engaged in any exercise after the termination of the training programs.

### 3.1. Primary Outcome

In both study groups, the mean and maximum menstrual pain intensity decreased significantly after the interventions (*p* < 0.05). There were also no significant differences within the groups in terms of the post-intervention and one-month follow-up values (*p* > 0.05).

According to intergroup comparisons, the PNE+ET group exhibited a greater decrease in menstrual pain intensity compared to the BPE+ET group following the intervention and at the one-month follow-up (*p* < 0.05, Cohen’s d = 0.68–1.17) (Table 2).

### 3.2. Secondary Outcomes

In both study groups, the scores on all the secondary outcomes decreased significantly after the interventions compared to the baseline (*p* < 0.05). There were no significant within-group differences in terms of the post-intervention and one-month follow-up values (*p* > 0.05).

According to the intergroup comparisons, the severity of the central sensitization symptoms and pain catastrophizing decreased more in the PNE+ET group compared to the BPE+ET group (*p* < 0.05, Cohen’s d = 0.725–0.804), while no differences between the groups were observed in terms of menstrual stress level (*p* > 0.05) (Table 2).

## 4. Discussion

To the best of our knowledge, this is the first study to address the effects of PNE or BPE in individuals with PD. The study found that PNE was more effective in improving menstrual pain, central sensitization-related symptoms, and menstrual pain catastrophizing compared to BPE after the intervention period and at the 1-month follow-up. However, the menstrual stress levels exhibited comparable changes.

Exercise interventions have been proposed as an alternative to analgesic agents in the management of PD. Various types of exercise have been demonstrated to be more effective than a placebo, including relaxation and stretching exercises [39,55]. The research on stretching exercises in PD has compared specific and/or general stretching exercises (either alone or in combination with diaphragmatic breathing, running, Kegel exercises, or menstrual care) with various interventions or with a control group [13,40,42,43,58,59]. The discrepancies in these study results can be attributed to the heterogeneity of the muscles being stretched, the exercise types, and the study protocols.

There is a paucity of studies on the use of diaphragmatic breathing as a relaxation intervention for participants suffering from PD. The existing studies incorporated diaphragmatic breathing into other interventions, such as a physiotherapy program or a progressive muscle relaxation technique, and showed that it improved menstrual pain, anxiety, quality of life, participation in social activities, and work/school performance compared to the non-intervention control group [42,60].

In both biomedical and biopsychosocial models, educating patients about their disease or pain is a key component of therapeutic motivation, adherence, and satisfaction [14,17].

As a conventional pain education approach, BPE can provide significant clinical benefits for injuries, in post-surgical settings, and in the acute phase of diseases. However, conventional methods are inadequate in managing complex processes associated with chronic pain. These processes encompass phenomena such as peripheral and central sensitization, facilitation and inhibition mechanisms, as well as immune and endocrine responses [18,20]. Furthermore, BPE may negatively affect treatment outcomes by increasing participants’ fear and stress [20].

Contemporary pain concepts extend beyond the scope of nociceptive (associated with tissue damage) or neuropathic (associated with nerve damage) factors. These concepts prioritize the role of the brain’s interpretation of pain, central sensitization, diminished modulation, and immune and endocrine activation [17]. PNE offers an explanation for biologically unexplained pain and other symptoms based on neurobiological mechanisms and facilitates the healing process [17,20]. PNE has been demonstrated to influence the emotional aspects of pain [17], and mitigate threats and catastrophizing by modifying beliefs and perceptions about pain [61,62] and enhancing pain coping [18]. Furthermore, there is evidence that PNE can lead to enhanced improvements in pain, movement, and fear-avoidance behaviors when supported by active strategies that promote a gradual return to normal activity and address sleep problems [20].

In the case of fibromyalgia, chronic pain following breast cancer surgery, chronic spinal pain, and chronic shoulder pain, the combination of PNE and exercises or standard physiotherapy has been reported to be more effective in reducing pain intensity compared to BPE or control interventions, such as exercise or standard physiotherapy alone [16,21,31,34,63]. Conversely, PNE or BPE, either as a stand-alone modality or combined with other interventions, have yielded similar outcomes in patients experiencing post-surgical or musculoskeletal pain [30,32,33,35,37].

Pain in primary dysmenorrhea (PD) can present in multiple forms, including physical, psychological, and behavioral symptoms [50,51,64]. In participants with PD, stretching exercises, whether performed alone or in combination with other interventions, have been reported to be more effective in alleviating menstrual symptoms compared to other interventions (e.g., heat application, abdominal massage) or the control group (no intervention or menstrual care) [13,40,42,58]. In the present study, it was observed that menstrual stress levels exhibited comparable declines with BPE and PNE combined with ET. This result may be due to the fact that the MDQ evaluates a multitude of additional symptom categories in addition to pain (e.g., water retention, autonomic reactions, concentration, etc.) that may be comparatively less affected by biopsychosocial factors.

Dysmenorrhea has been identified as a potential risk factor for the development of central sensitization and chronic pain. In cases of repetitive cyclic pain, there is a possibility of peripheral sensitization, which can lead to alterations in the corticolimbic system. This, in turn, can trigger abnormal stress responses through the hypothalamus–pituitary–adrenal axis. Over time, these changes may result in viscero–visceral and visceral–somatic interactions, potentially transitioning from acute pain to chronic pain [6,7]. It is imperative to acknowledge the profound interplay between the underlying biological mechanisms and psychosocial factors in diseases and pain that are associated with central sensitization. Consequently, the management of these conditions necessitates an approach that is grounded in a biopsychosocial framework [7,36]. In the present study, we found that the combination of PNE and exercise training resulted in a substantial improvement in central sensitization symptoms after the intervention and a moderate improvement at the short-term follow-up compared to BPE in individuals with PD. The findings of this study are consistent with the results of previous studies indicating that a combination of PNE and physiotherapy or exercise leads to a reduction in the severity of central sensitization symptoms or pressure pain sensitivity at local and/or remote areas compared with BPE or control interventions (exercise or standard physiotherapy) in individuals experiencing pain following breast cancer surgery and in those with chronic spinal pain [16,21,30,31]. Cyclical pain in PD is not limited to peripheral changes; it also leads to changes in pain processing in the central nervous system. It has been reported that connections between the PAG and the dorsolateral prefrontal cortex (DLPFC), which are involved in descending pain modulation, are weakened, which may lead to inadequate pain inhibition and central sensitization [65]. These changes, which point to visceral hypersensitivity, strengthen the rationale for the use of the CSI in individuals with PD. Furthermore, central sensitization should be considered as being mediated not only by neurophysiological, but also by neuroimmune pathways, and that increased inflammatory cytokine levels and stress responses may affect central pain processing [6,19,24].

Pain catastrophizing is defined as a negative cognitive orientation toward pain [53]. The tendency to catastrophize menstrual pain, particularly the feelings of powerlessness and helplessness that accompany recurrent menstrual pain, is a significant psychological factor associated with menstrual pain [66]. In the present study, we found that the combination of PNE and ET resulted in a substantial reduction in pain catastrophizing after the intervention and a moderate reduction at the short-term follow-up compared to BPE in individuals with PD. This result is consistent with the hypothesis that PNE, when administered as a stand-alone treatment or in combination with exercise or a standard physiotherapy program, is more efficacious in reducing pain catastrophizing than BPE or control interventions (exercise or standard physiotherapy) in populations experiencing musculoskeletal or surgical pain [16,31,32,33]. Conversely, some studies have reported that PNE combined with physiotherapy or exercise interventions provides improvements in pain catastrophizing similar to those of BPE [21,34,35]. The variations in study outcomes can be attributed to differences in the study populations, pain severity, pain type, and intensity of pain education [21,34,35,37].

The strengths of the present study include the parallel-group randomized design, inclusion of a follow-up period, and the utilization of widely used, reliable, and valid outcome measurement tools. Considering the limited efficacy and adverse effects of current pharmacologic agents in PD, the study provides evidence for the combined effect of non-pharmacologic approaches in PD, a common gynecologic condition. In addition, the low cost and easy implementation of the interventions can be considered another strength that could accelerate their dissemination, especially in resource-limited clinical settings.

It is important to note that none of the participants dropped out of the study. In addition, while the participants showed full compliance with the exercise and pain education sessions in the clinic (100%), the compliance rates for the exercise sessions as a home program were also quite high (PNE+ET: 97.70 ± 4.27%; BPE+ET: 97.04 ± 5.65%). One factor for the high compliance may be the fact that patient and/or pain education, which are mostly neglected in disease/pain management, were provided in this study and have been shown to increase motivation and compliance with treatment [22]. Another factor may have been the reasonable number of face-to-face exercises (once a week) and education sessions, and the fact that the researchers tried to keep the motivation of all the participants high during the study period. The number of face-to-face intervention sessions was kept low, and a home program was provided to prevent participants from dropping out of the study.

One study limitation is that due to the nature of the interventions and the self-reported outcome measures used in the study, it was not possible to blind the participants to the interventions. However, the absence of the researcher implementing the interventions during the evaluation and blinding the observing clinician to the intervention were important factors in reducing bias. Another limitation of the study is the one-month follow-up period. A longer follow-up would be needed to assess the sustainability of the intervention’s effects and should be addressed in future research. Although pain and its related symptoms were assessed using self-report scales with proven reliability and validity, the results should be verified using objective biochemical markers and imaging methods (e.g., USG). Since a more intensive PNE program is more effective than a lower intensity PNE program in individuals with fibromyalgia [63], more intensive pain education programs for individuals with PD should be investigated in future studies. In addition, beyond pain education sessions, communication and supervision consistent with the PNE philosophy during exercise and relaxation sessions may further enhance the potential of PNE. Although various lifestyle characteristics important for PD (smoking, alcohol consumption, and regular exercise habits) were considered in our study, it is also important to consider the nutritional habits and dietary characteristics of the participants in further studies.

## 5. Conclusions

In individuals diagnosed with PD, PNE, in combination with stretching and relaxation exercises, was shown to be more efficacious in reducing menstrual pain intensity, the severity of somatic and emotional symptoms associated with central sensitization, and pain catastrophizing compared to BPE. Further studies are needed to assess the efficacy of pain education programs of different intensities in combination with diverse conservative interventions in individuals with PD. Accordingly, clinicians could consider using PNE in conjunction with exercise-based approaches in the management of pain and its related psychological symptoms in PD.

## Figures and Tables

**Table 1:** Baseline characteristics of participants.

Characteristic	PNE+ET (n = 19)	BPE+ET (n = 19)	*p*-Value
**Demographic and physical characteristics**			
**Age, y**	20.89 ± 3.51	20.37 ± 2.36	0.591 ^a^
**Education**			
**˂High school**	-	-	1.000 ^b^
**≥High school**	19 (100%)	19 (100%)	
**Marital status**			
**Single**	19 (100%)	19 (100%)	1.000 ^b^
**Married**	-	-	
**Working status**			
**Employed**	3 (15.8%)	2 (10.5%)	0.500 ^b^
**Unemployed**	16 (84.2%)	17 (89.5%)	
**BMI, kg/m^2^**	22.50 ± 3.20	22.56 ± 3.60	0.956 ^a^
**Lifestyle characteristics**			
**Smoking**			
**No**	16.0 (84.2%)	14.0 (73.7%)	0.426 ^b^
**Yes**	3.0 (15.8%)	5.0 (26.3%)	
**Smoking exposure, package-year**	3.09 ± 3.43	0.92 ± 1.21	0.180 ^a^
**Alcohol consumption**			
**No**	18.0 (94.7%)	18.0 (94.7%)	1.000 ^b^
**Yes**	1.0 (5.3%)	1.0 (5.3%)	
**Regular exercise**			
**No**	19.0 (100%)	19.0 (100%)	1.000 ^b^
**Yes**	-	-	
**Menstrual characteristics**			
**Age at menarche, y**			
**<12 y**	1 (5.3%)	-	0.311 ^b^
**≥ 12 y**	18 (94.7%)	19 (100%)	
**Menstrual cycle duration, d**	27.11 ± 3.25	28.68 ± 3.23	0.234 ^c^
**Menstruation duration, d**			
**3–7 d**	19 (100%)	19 (100%)	1.000 ^b^
**>7 d**	-	-	
**Average menstrual pain severity in the last 6 months**	6.71 ± 1.66	6.35 ± 1.64	0.593 ^c^
**Menstrual pain duration, hours/cycle**	13.84 ± 15.81	16.74 ± 17.49	0.525 ^c^
**Use of medication (analgesic, NSAI) for PD**			
**No**	10 (52.6%)	12 (63.2%)	0.511 ^b^
**Yes**	9 (47.4%)	7 (36.8%)	
**Use of analgesic/NSAI during last menstruation**			
**No**	10 (52.6%)	13 (68.4%)	0.319 ^b^
**Yes**	9 (47.4%)	6 (31.6%)	

Data are presented as mean ± standard deviation or number (percentage). ET: exercise training; PNE: pain neuroscience education; BPE: biomedical pain education; n: number; BMI: body mass index; *p* ^a^: independent groups *t*-test; *p* ^b^: Fisher–Freeman–Halton exact test; *p* ^c^: Mann–Whitney U test.

**Table 2:** Comparison of primary and secondary outcome measures within and between groups.

Outcome Measure	Time Point	Within-Group Effect		Between-Group Effect	Between-Group Effect Size	Cohen’s d 95% CI	
		PNE+ET (n = 19)	BPE+ET (n = 19)	*p* ^a^	d_Cohen_	*Lower*	*Upper*
**Primary Outcome Measures**							
Mean pain intensity, cm	Baseline	4.74 ± 1.36 **^x^**	4.64 ± 1.95 **^x^**	0.863			
	After	1.70 ±0.96 ^y^	2.93 ± 1.82 ^y^	***0.023***	0.847	−1.507	−0.177
	Follow-up	1.85 ± 1.07 **^y^**	3.23 ± 1.28 **^y^**	***0.001***	1.174	−1.858	−0.477
	*p* ^b^	**<0.001**	**0.002**				
Maximum pain intensity, cm	Baseline	7.74 ± 1.29 ^x^	7.09 ± 1.5 ^x^	0.339			
	After	3.91 ± 1.72 ^y^	5.24 ± 1.67 ^y^	***0.030***	0.786	−1.442	−0.120
	Follow-up	4.18 ± 2.24 ^y^	5.48 ± 1.48 ^y^	***0.046***	0.683	−1.333	−0.023
	*p* ^b^	**<0.001**	**0.003**				
**Secondary Outcome Measures**							
Menstrual stress	Baseline	77.89 ± 25.10 ^x^	80.37 ± 30.73 ^x^	0.751			
	After	40.05 ± 23.02 ^y^	46.21 ± 27.09 ^y^	0.525	0.245	−0.882	0.395
	Follow-up	36.21 ± 26.39 ^y^	44.11 ± 30.90 ^y^	0.325	0.275	−0.912	0.366
	*p* ^b^	**<0.001**	**<0.001**				
Central sensitization symptoms	Baseline	43.05 ± 19.05 ^x^	36.37 ± 20.29 ^x^	0.418			
	After	18.16 ± 10.38 ^y^	31.00 ± 20.07 ^y^	***0.043***	0.804	−1.461	−0.136
	Follow-up	16.95 ± 10.34 ^y^	29.11 ± 20.15 ^y^	***0.043***	0.759	−1.414	−0.095
	*p* ^b^	**<0.001**	**0.046**				
Menstrual pain catastrophizing	Baseline	25.26 ± 12.80 ^x^	24.05 ± 15.46 ^x^	0.773			
	After	7.37 ± 5.19 ^y^	15.53 ± 13.51 ^y^	***0.043***	0.797	−1.454	−0.130
	Follow-up	7.42 ± 7.46 ^y^	15.53 ± 13.94 ^y^	***0.025***	0.725	−1.378	−0.063
	*p* ^b^	**<0.001**	**<0.001**				

Data are presented as mean ± standard deviation. ET: exercise training; PNE: pain neuroscience education; BPE: biomedical pain education; n: number; d**_Cohen_**: Cohen’s d between-group effect size; CI: confidence interval; *p* ^a^: Mann–Whitney U test (between-group comparison); *p* ^b^: Friedman test (within-group comparison); ^x,y^: according to the Conover post hoc test, different upper indices in the same column indicate within-group differences between time points.

**Table 3:** Compliance to clinical and home exercise sessions of groups.

Exercise Compliance	PNE + ET (*n* = 19)	BPE + ET (*n* = 19)	*p*
Exercise compliance (%)	97.70 ± 4.27 87.50–100%	97.04 ± 5.65 81.25–100%	0.895

Data are presented as mean ± standard deviation (min-max). ET: exercise training; PNE: pain neuroscience education; BPE: biomedical pain education; n: number; *p*: Mann–Whitney U test (between-group comparison).

## Data Availability

The raw data supporting the conclusions of this article will be made available by the authors on request.
